# Factors associated with liver cancer prognosis after hepatectomy: A retrospective cohort study

**DOI:** 10.1097/MD.0000000000027378

**Published:** 2021-10-22

**Authors:** Yutao Yuan, Fangnian Yang, Yuanyuan Wang, Yusong Guo

**Affiliations:** a Department of Clinical Laboratory, Xiamen Haicang Hospital, Xiamen, P.R. China; b Department of Blood Transfusion, Zhongshan Hospital Affiliated to Xiamen University, Xiamen, P.R. China.

**Keywords:** liver cancer, prognosis, progression, risk factor

## Abstract

This article was to investigate risk factors influencing liver cancer prognosis after hepatectomy.

Patients undergoing hepatectomy after being diagnosed with liver cancer in Zhongshan Hospital Affiliated to Xiamen University were collected in the retrospective cohort study between January 2012 and December 2017, and divided into disease progression and non-progression groups based on their prognostic status. Univariate analysis was performed on the patients’ baseline and laboratory test data, with multivariate logistic regression further conducted to investigate the independent risk factors for liver cancer progression after hepatectomy.

Among the 288 subjects, 159 had adverse outcomes (death or cancer recurrence). Hepatitis B and high levels of aspartate aminotransferase, gamma-glutamyltransferase, alkaline phosphatase (ALP), direct bilirubin, and total bilirubin as well as low level of lymphocyte (LYM) were found to be associated with disease progression in the univariate analysis, and were introduced into the multivariate logistic regression. The results indicated that patients with high ALP level (odds ratio [OR] = 1.004, 95%CI: 1.002–1.007, *P* = .003) and with a history of hepatitis B (OR = 2.182, 95%CI: 1.165–4.086, *P* = .015) had a higher risk of liver cancer progression compared with those of lower ALP level and those without hepatitis B respectively, whereas the elevated level of LYM (OR = 0.710, 95%CI: 0.516–0.978, *P* = .034) had favorable progression.

The elevated ALP level and a history of hepatitis B may increase the risk of death or cancer recurrence, whereas high LYM level may decrease poor progression among liver cancer patients after hepatectomy. More importance should be attached to the improvement of the liver function and treatment of hepatitis B to enable a better outcome for the patients.

## 1 Introduction

Liver cancer remains the sixth most common cancer around the world and the second leading cause of cancer related mortality.^[1]^ The mortality of liver cancer patients is very high, and there are nearly 800,000 deaths annually all over the world.^[2]^ Among the 5 most lethal cancers in the United States, the mortality of liver cancer increases faster than other cancers each year,^[3]^ and the occurrence of liver cancer is even more frequent in developing countries.^[4]^ Nowadays, liver cancer has become a global problem gradually. The occurrence of liver cancer can be associated with the infection of chronic hepatitis B virus (HBV) in the presence or absence of cirrhosis.^[5]^ Other risk factors for liver cancer may include liver cirrhosis, metabolic disorders, excessive drinking, certain genetic diseases, exposure to aflatoxins, and etc.^[6]^

For liver cancer patients at different stages, the treatments are different. Patients of the early stage of liver cancer should receive hepatectomy, transplantation, radiofrequency ablation, while those with advanced stages receive transcatheter arterial chemoembolization and chemotherapy.^[7–10]^ Despite the significant advances in the diagnosis and treatment that have been achieved in recent years, its curative effect is still not satisfactory. The 5-year postoperative survival rate of patients with liver cancer is 37% to 65%, whereas the proportion of recurrence is as high as 75% to 100%.^[11–13]^

In the present study, we retrospectively collected laboratory and survival data of patients with liver cancer admitted to our department and evaluated the influencing factors for adverse outcomes after hepatectomy through univariate and multivariate analyses, in order to identify patients at high risks of death or recurrence at an early stage.

## 2 Methods

### 2.1 Patient enrollment

Patients were conducted pre-surgical workups including tumor markers, physical examination, liver function test, and blood chemistry evaluation according to admission requirements of our hospital. The Affiliated Hospital of Xiamen University is a large-scale comprehensive Grade 3A hospital, which has made great contributions to the construction of a research-oriented hospital affiliated to the national key university.

Patients undergoing hepatectomy after being diagnosed with liver cancer in Zhongshan Hospital Affiliated to Xiamen University Hospital were enrolled in the current study from January 2012 to December 2017, and who met any of the following criteria were excluded: history of liver transplantation; death during the main hepatectomy; extrahepatic metastasis during hepatectomy; residual hepatocellular carcinoma (HCC) after hepatectomy; those with death or recurrence of liver cancer after hepatectomy till December 2017 were divided into the disease progression group (cases), and others were into the non-progression group (controls).

This study was approved by the Ethics Committee of Zhongshan Hospital Affiliated to Xiamen University [No. xmzsyyky (2019043)] in accordance with the Declaration of Helsinki and National Clinical Research regulations.

### 2.2 Data collection

Data that needed to be collected included the patients’ gender, age, length of stay in hospital, time to cancer progression, Child-Pugh classification, hematocrit value, red blood cell distribution width-standard deviation, red blood cell distribution width-coefficient of variation, prothrombin time, activated partial thromboplastin time and levels of alpha-fetoprotein, platelet, albumin, carcinoembryonic antigen, carbohydrate antigen 125, carbohydrate antigen 199, hemoglobin, white blood cell, neutrophil, lymphocyte (LYM), monocyte, fibrinogen, alanine transaminase, aspartate aminotransferase (AST), gamma-glutamyltransferase (GGT), alkaline phosphatase (ALP), direct bilirubin (DBIL), indirect bilirubin, and total bilirubin (TBIL). Information regarding if the patient had hypertension, hepatitis B, liver cirrhosis, blood transfusions, infections, complications, ascites, and adverse outcomes was also recorded.

### 2.3 Follow-up

Patients were followed up every 3 to 4 months after hepatectomy until death or December 2017, and the physical examination, liver function test, blood chemistry evaluation, and dynamic contrast-enhanced computed tomography were performed during each follow-up.

Patients meeting one of the following criteria were diagnosed as recurrence of liver cancer: observed HCC on dynamic contrast-enhanced computed tomography, dynamic contrast-enhanced magnetic resonance imaging, or hepatic angiography; recurrence evidence after liver biopsy or re-hepatectomy.

### 2.4 Statistical analysis

The data were analyzed using SPSS statistical software (SPSS Inc., Chicago, IL). The normally distributed quantitative data were expressed as mean ± standard deviation (±s), and assessed by *t* test (Student *t* test). The quantitative data of skewed distribution were presented as median and quartiles [M (Q_1_, Q_3_)], and rank sum test (Mann–Whitney *U* test) was performed. The ordinal data were displayed as number of cases and percentiles N (%), and also examined by Mann–Whitney *U* test. Categorical data were expressed as number of cases and percentiles N (%), and Pearson chi-squared test was conducted when the total number of cases n ≥ 40 and the theoretical frequency T ≥ 5, while when n < 40 or T < 5, Fisher exact probability method was used. Statistically significant variables in the univariate analysis were then included in the multivariate logistic regression to further explore the factors that influenced the prognosis of liver cancer patients. *P* < .05 was considered of statistical significance.

## 3 Results

### 3.1 Analysis of baseline characteristics of included patients

Initially, 297 subjects were included in the present study, removing 9 patients belonging to Child-Pugh C, of totally 288 which 159 had adverse outcomes (death or recurrence of liver cancer), accounting for 55.21%. The average age was 54.92 years (SD of ±13.52 years), and the mean length of stay in hospital was 14.5 (10.0, 20.0) days. The constituent ratio of patients with hepatitis B was higher in the progression group than that in the non-progression group (χ^2^ = 4.174, *P* = .041). No significant differences in gender, age, hypertension, liver cirrhosis, blood transfusions, infections, complications, ascites, and length of stay in hospital were observed between the 2 groups, with all *P* > .05 (Table 1).

**Table 1:** Comparison of baseline information between 2 groups.

		Groups			
Variables	Description	Non-progression group (n = 129)	Progression group (n = 159)	Statistics	*P*
Gender, n (%)				χ^2^ = 0.186	.666
Male	239 (80.20)	110 (79.14)	129 (81.13)		
Female	59 (19.80)	29 (20.86)	30 (18.87)		
Age (yrs)	54.92 ± 13.52	54.28 ± 12.79	55.44 ± 14.11	t = –0.72	.473
Hypertension, n (%)				χ^2^ = 0.114	.736
No	241 (83.68)	109 (84.50)	132 (83.02)		
Yes	47 (16.32)	20 (15.50)	27 (16.98)		
Hepatitis B, n (%)				χ^2^ = 4.174	.041
No	100 (34.72)	53 (41.09)	47 (29.56)		
Yes	188 (65.28)	76 (58.91)	112 (70.44)		
Liver cirrhosis, n (%)				χ^2^ = 0.136	.712
No	164 (56.94)	75 (58.14)	89 (55.97)		
Yes	124 (43.06)	54 (41.86)	70 (44.03)		
Blood transfusion, n (%)				χ^2^ = 0.638	.424
No	226 (78.47)	104 (80.62)	122 (76.73)		
Yes	62 (21.53)	25 (19.38)	37 (23.27)		
Infection, n (%)				χ^2^ = 1.778	.182
No	258 (89.58)	119 (92.25)	139 (87.42)		
Yes	30 (10.42)	10 (7.75)	20 (12.58)		
Ascites, n (%)				Fisher	.695
No	282 (97.92)	127 (98.45)	155 (97.48)		
Yes	6 (2.08)	2 (1.55)	4 (2.52)		
Complication, n (%)				χ^2^ = 1.071	.301
No	255 (88.54)	117 (90.70)	138 (86.79)		
Yes	33 (11.46)	12 (9.30)	21 (13.21)		
Hospital stay (days), [M (Q_1_, Q_3_)]	14.5 (10.0, 20.0)	14.0 (10.0, 20.0)	15.0 (10.0, 21.0)	Z = –0.702	.483

### 3.2 Comparison of laboratory test results after hepatectomy

As shown in the Table 2, the progression group had a larger number of patients belonging to Child-Pugh class C compared with the non-progression group (χ^2^ = 4.260, *P* = .039). There were higher levels of AST (Z = –2.952, *P* = .003), GGT (Z = –4.707, *P* < .001), LYM (Z = 2.046, *P* = .041), ALP (Z = –3.205, *P* = .001), TBIL (Z = –2.794, *P* = .005), and DBIL (Z = –3.353, *P* < .001) found in the cases than in the controls. All the differences mentioned above were statistically significant. However, the differences in alpha-fetoprotein, carcinoembryonic antigen, carbohydrate antigen 125, carbohydrate antigen 199, hemoglobin, hematocrit, platelet, red blood cell distribution width-standard deviation, red blood cell distribution width-coefficient of variation, white blood cell, neutrophil, monocyte, prothrombin time, activated partial thromboplastin time, fibrinogen, albumin, alanine transaminase, and indirect bilirubin levels between the 2 groups had no statistical significance (all *P* > .05).

**Table 2:** Comparison of laboratory tests between 2 groups.

		Groups			
Variables	Description	Non-progression group (n = 129)	Progression group (n = 159)	Statistics	*P*
Child-Pugh classification, n (%)				χ^2^ = 4.260	.039
B	185 (92.04)	96 (96.00)	89 (88.12)		
C	16 (7.96)	4 (4.00)	12 (11.88)		
AFP (ng/mL), [M (Q_1_, Q_3_)]	108.50 (16.20, 2253.50)	133.30 (18.14, 1411.00)	99.96 (15.29, 2553.00)	Z = –0.005	.996
CEA (ng/mL), [M (Q_1_, Q_3_)]	3.06 (0.84, 4.10)	3.07 (2.76, 3.37)	2.13 (0.77, 4.83)	Z = 0.167	.868
CA125 (U/mL), [M (Q_1_, Q_3_)]	19.65 (13.95, 28.90)	24.00 (24.00, 24.00)	15.30 (12.60, 33.80)	Z = 0.001	1.000
CA199 (U/mL), [M (Q_1_, Q_3_)]	21.60 (17.30, 49.50)	21.60 (21.60, 21.60)	33.40 (14.45, 485.40)	Z = 0.001	1.000
Hb (g/dL), [M (Q_1_, Q_3_)]	118.74 ± 18.79	118.19 ± 17.91	119.20 ± 19.54	t = –0.45	.656
HCT (%), [M (Q_1_, Q_3_)]	35.20 ± 6.11	34.97 ± 5.02	35.38 ± 6.89	t = –0.57	.568
PLT (10^9^/L), [M (Q_1_, Q_3_)]	159.00 (112.00, 215.00)	170.00 (108.00, 220.00)	153.00 (112.00, 209.00)	Z = 0.953	.340
RDW_SD (fL), [M (Q_1_, Q_3_)]	46.31 ± 8.20	46.23 ± 6.59	46.37 ± 9.36	t = –0.15	.883
RDW_CV (%), [M (Q_1_, Q_3_)]	13.60 (12.80, 14.90)	13.50 (12.70, 14.80)	13.70 (12.90, 15.10)	Z = –0.825	.410
WBC (10^9^/L), [M (Q_1_, Q_3_)]	7.61 (5.93, 9.55)	7.52 (5.76, 9.99)	7.76 (6.04, 9.39)	Z = –0.589	.556
NEUT (%), [M (Q_1_, Q_3_)]	5.31 (4.02, 7.04)	5.21 (3.80, 6.85)	5.43 (4.18, 7.11)	Z = –0.744	.457
LYM (10^9^/L), [M (Q_1_, Q_3_)]	1.16 (0.85, 1.61)	1.23 (0.89, 1.68)	1.11 (0.80, 1.55)	Z = 2.046	.041
MONO (10^9^/L), [M (Q_1_, Q_3_)]	0.62 (0.43, 0.83)	0.61 (0.43, 0.81)	0.62 (0.43, 0.85)	Z = –0.735	.463
PT (s), [M (Q_1_, Q_3_)]	14.50 (13.40, 15.80)	14.20 (13.30, 15.30)	14.70 (13.50, 15.95)	Z = –1.133	.257
APTT (s), [M (Q_1_, Q_3_)]	35.50 (29.80, 40.30)	34.30 (28.80, 39.60)	36.25 (31.20, 40.75)	Z = –1.620	.105
FIB (g/L), [M (Q_1_, Q_3_)]	3.17 (2.27, 4.32)	3.13 (2.33, 4.21)	3.19 (2.16, 4.49)	Z = –0.032	.975
ALB (g/L), [M (Q_1_, Q_3_)]	34.35 ± 5.17	34.27 ± 5.51	34.41 ± 4.89	t = –0.22	.826
ALT (U/L), [M (Q_1_, Q_3_)]	73.00 (43.50, 128.00)	65.50 (43.00, 119.00)	86.80 (45.20, 131.00)	Z = –1.579	.114
AST (U/L), [M (Q_1_, Q_3_)]	47.15 (30.40, 93.50)	40.30 (28.70, 81.00)	50.70 (34.70, 119.00)	Z = –2.952	.003
GGT (U/L), [M (Q_1_, Q_3_)]	90.75 (54.00, 165.30)	71.70 (48.00, 113.30)	111.00 (68.50, 198.00)	Z = –4.707	<.001
ALP (U/L), [M (Q_1_, Q_3_)]	98.00 (71.85, 153.50)	93.00 (67.10, 118.00)	110.50 (76.50, 215.00)	Z = –3.205	.001
TBIL (μmol/L), [M (Q_1_, Q_3_)]	16.98 (11.95, 24.75)	15.75 (11.24, 21.60)	18.15 (12.70, 31.40)	Z = –2.794	.005
DBIL (μmol/L), [M (Q_1_, Q_3_)]	7.87 (5.60, 11.95)	7.15 (5.15, 9.35)	9.10 (5.80, 16.15)	Z = –3.353	<.001
IBIL (μmol/L), [M (Q_1_, Q_3_)]	8.65 (6.10, 12.40)	8.37 (5.60, 11.95)	9.55 (6.25, 13.00)	Z = –1.338	.181

### 3.3 Multiple logistic regression analysis

Variables with significant differences in the univariate analysis were introduced into the multivariate logistic regression, including hepatitis B, Child-Pugh classifications, LYM, AST, GGT, ALP, TBIL, and DBIL, to find out the risk factors for adverse outcomes among the patients. High ALP level and a history of hepatitis B were factors influencing the prognosis of liver cancer patients after hepatectomy. Patients with a history of hepatitis B had a higher risk of 1.182-fold for progression than those without hepatitis B (odds ratio [OR] = 2.182, 95%CI: 1.165–4.086, *P* = .015). Elevated ALP level after hepatectomy was associated with poor prognosis of patients (OR = 1.004, 95%CI: 1.002–1.007, *P* = .003). In addition, for every 1 unit increased in LYM after hepatectomy, the probability of progression was decreased by 0.290 times (OR = 0.710, 95%CI: 0.516–0.978, *P* = .034) (Table 3).

**Table 3:** Factors affecting the HCC prognosis after hepatectomy.

						*95%CI*	
Variables	*β*	*SE*	*Wald*	*P*	*OR*	Lower	Upper
ALP (U/L)	0.004	0.001	9.039	.003	1.004	1.002	1.007
LYM (10^9^/L)	−0.342	0.163	4.397	.036	0.710	0.516	0.978
Hepatitis B	0.390	0.160	5.937	.015	2.182	1.165	4.086

## 4 Discussion

The prognosis of patients with liver cancer is not optimistic. Yet, to know the prognostic factors after hepatectomy is of vital importance for reducing the subsequent mortality and the potential risks for recurrence. In our study, the elevated ALP and LYM levels as well as a history of hepatitis B were found to be closely related to the progression of liver cancer. Among these factors, the high ALP level and a history of hepatitis B were independent risk factors, whereas high LYM counts was a protective factor of the poor prognosis in HCC patients after hepatectomy.

ALP, as one of serum liver enzymes, is routinely tested after surgery and is easy to obtain from the liver, bile duct, bone, and so on, which is considered as a significant adverse prognostic indicator.^[14]^ The ALP level has been widely used to evaluate the liver function and to predict prognosis in patients with liver cancer after treatment.^[15–17]^ Yeh et al^[18]^ pointed out that the elevated ALP level played an important role in the prognosis of HCC patients at intermediate stage. The study conducted by Wu et al^[14]^ demonstrated that the high serum ALP level could reflect the overburden of tumors or the occurrence of metastasis, suggesting that liver cancer patients had the poor prognosis. This finding was in line with our results that liver cancer patients with high ALP level had a high risk of progression.

The occurrence and development of liver cancer are related to the abnormal changes of immune function status.^[19]^ Immune cells, such as LYM counts that reflect the body immune function and participate in the anti-hepatoma immune response.^[19]^ Normal immune status is the key mechanism of the anti-hepatoma status.^[19]^ Studies have suggested that abnormal immune status is not only involved in the occurrence of tumors, but also may relative with the metastasis and recurrence of tumors.^[20]^ However, some scholars mentioned that most of single immunological indicator may not have a strong power for predicting the survival and recurrence of HCC patients after hepatectomy.^[21]^ Future study still need to validate the result.

HBV is the main pathogen that infects the liver.^[22]^ There is an epidemiological relationship between the infection of HBV and the progression of liver cancer.^[23]^ HBV causes liver cancer in both direct and indirect ways, and can accelerate the development of liver cancer.^[24]^ The role of HBV in the development of liver cancer has been investigated in many studies: HBV integrates their DNA into the host genome, causing mutations and chromosomal instability.^[25,26]^ HBV interacts with host cell molecules directly to influence the vital hepatocyte functions, such as proliferation, apoptosis, migration, and so on.^[27,28]^ HBV may promote the carcinogenic action via stimulating inflammatory responses.^[29]^ Maucort-Boulch et al^[30]^ revealed that about two thirds of liver cancer cases in less developed countries were caused by HBV, along with a quarter in more developed countries. The suppression of HBV with conventional anti-viral therapy alone is not sufficient to prevent the development of liver cancer, and future treatment may be combined with the targeted covalently closed circular DNA, inhibited entry of virus into the newly formed hepatocytes and the T-cell vaccination.^[31]^

There are still some limitations in the current study: A relatively small sample size might weaken the statistical power of the study. All the data included were retrospectively collected, and more multicenter, large-scale, and prospective studies are called for to explore effective treatment models.

## 5 Conclusions

This study indicated that elevated ALP level and a history of hepatitis B were independent risk factors, whereas high LYM counts was a protective factor of the poor prognosis in HCC patients after hepatectomy. Regular follow-up for the detection of early recurrence and further treatments on improving liver function or HBV cure are needed in postoperative liver cancer patients.

## Author contributions

YY and YG designed the study. YY wrote the manuscript. YW collected, analyzed and interpreted the data. FY analyzed and interpreted the data, and critically edited the manuscript. YG critically reviewed and approved the manuscript. All authors read and approved the final manuscript.

**Conceptualization:** Yutao Yuan, Yusong Guo.

**Data curation:** Fangnian Yang, Yuanyuan Wang.

**Formal analysis:** Yuanyuan Wang.

**Writing – original draft:** Yutao Yuan.

**Writing – review & editing:** Fangnian Yang, Yuanyuan Wang, Yusong Guo.
